# Immunohistochemical Expression of p53 and FGFR3 Predicts Response to Enfortumab Vedotin in Metastatic Urothelial Carcinoma

**DOI:** 10.3390/ijms251910348

**Published:** 2024-09-26

**Authors:** Yujiro Nagata, Akinori Minato, Hisami Aono, Rieko Kimuro, Katsuyoshi Higashijima, Ikko Tomisaki, Kenichi Harada, Hiroshi Miyamoto, Naohiro Fujimoto

**Affiliations:** 1 Department of Urology, School of Medicine, University of Occupational and Environmental Health, Kitakyushu 807-8555, Japan; 2 Departments of Pathology & Laboratory Medicine and Urology, University of Rochester Medical Center, Rochester, NY 14642, USA; 3 Department of Urology, Kurate Hospital, Kurate 807-1311, Japan

**Keywords:** urothelial carcinoma, bladder cancer, upper urinary tract cancer, enfortumab vedotin, p53, FGFR3

## Abstract

Locally advanced or metastatic urothelial carcinoma is a genomically and molecularly heterogeneous disease associated with various clinical outcomes. We aimed to evaluate the association between the status of p53/FGFR3 expression and the efficacy of enfortumab vedotin (EV) in metastatic urothelial carcinoma. We evaluated the association between p53 (abnormal vs. wild-type) or FGFR3 (high vs. low) expression determined by immunohistochemistry and response to EV in 28 patients with metastatic urothelial carcinoma. Overall, 60.7% showed abnormal p53, and 17.9% had high FGFR3 expression. The rates of objective response to EV were statistically higher in patients with abnormal p53 than in those with wild-type p53 (*p* = 0.038). Patients with pure urothelial carcinoma (*n* = 18) and low FGFR3 showed significantly better response to EV than those with high FGFR3. When the statuses of p53 and FGFR3 were combined, abnormal p53/low FGFR3 (vs. wild-type p53/high FGFR3) was strongly associated with favorable outcomes in both the entire cohort (*p =* 0.002) and in cases of pure urothelial carcinoma only (*p* = 0.023). Immunohistochemically abnormal p53 tumors were found to respond well to EV, while high FGFR3 tumors had a poorer response. Thus, p53 and FGFR3 are potential biomarkers for predicting response to EV treatment in patients with urothelial carcinoma.

## 1. Introduction

The occurrence of urothelial carcinoma (UC) in the urinary bladder and upper urinary tract is increasing worldwide [1,2]. For decades, platinum-based chemotherapy has been the first-line standard for metastatic UC (mUC); however, treatment outcomes remain dismal, with a relatively low 5-year survival rate (e.g., 8.3% in bladder cancer) [3]. Recent advances with, for example, immune checkpoint inhibitors, antibody-drug conjugates, and a potent inhibitor of FGFR1–FGFR4, erdafitinib, have remarkably changed the therapeutic landscape of mUC. Despite such advances in the systemic treatment of mUC, the prognosis remains poor, and challenges persist in selecting optimal therapy, treatment sequences, and combination regimens. The further identification of biomarkers, as well as identifying key molecules or genes responsible for therapeutic susceptibility, may provide personalized approaches in patients with mUC.

Enfortumab vedotin (EV), an antibody–drug conjugate directed against nectin-4, demonstrated a survival benefit in patients with previously treated locally advanced UC or mUC, leading to its approval for the treatment of mUC [4]. An increasing body of real-world data in patients with advanced UC who received EV therapy has provided evidence for its efficacy comparable to that observed in a landmark clinical trial [5,6,7,8,9,10]. Nonetheless, the underlying mechanisms of intrinsic and acquired resistance to EV treatment are not yet fully understood, and analyses of potential biomarkers to predict response to EV in patients with UC are limited [11,12]. Moreover, EV-302, a phase 3, global, open-label, randomized trial comparing the efficacy and safety of combined EV and a programmed death-1 inhibitor, pembrolizumab, vs. those of platinum-based chemotherapy in patients with previously untreated locally advanced UC or mUC, demonstrated significant improvement in oncologic outcomes with the former [13]. This could be considered a landmark trial that has set a new standard. Consequently, this trial elicits several new questions that set the framework for future clinical and translational research, including how to identify UC patients who are likely to respond to EV and pembrolizumab. As EV is incorporated into the first-line setting, the development of predictive biomarkers for EV is critical to maximizing its clinical efficacy.

In recent years, genomic profiling has suggested that UC can be classified into several different molecular subtypes [14,15], which have distinct prognoses and vulnerabilities to chemotherapy or immunotherapy [16,17,18]. The *TP53* tumor-suppressor gene and the *FGFR3* oncogene are frequently mutated in muscle-invasive bladder cancer [14,19], and are included in seven key bladder cancer-associated genes for the consensus classes [19]. *TP53* represents the most frequently altered gene in the basal/squamous (Ba/Sq) subtype which corresponds to the Ba/Sq (TCGA group), Ba/Sq (Lund University group), and basal (MD Anderson group) subtypes [19]. *FGFR3* genomic alterations are enriched in luminal papillary (LumP) tumors (55%) which correspond to luminal-papillary (TCGA group) and UroA (Lund University group) subtypes [19]. Remarkably, *FGFR3* alterations are known to be more frequently observed in non-muscle-invasive UC than in muscle-invasive UC [20]. Meanwhile, bladder and upper tract UCs share various genomic alterations, such as those in *TP53* and *FGFR3*, but their frequency is different [20]. For example, compared with bladder UC (26%), *FGFR3* was more frequently altered in upper tract UC (40%) [21]. Of note, the association of molecular/genetic classification of UC with response to EV therapy has not been fully assessed, while EV has been approved as third-line therapy (after platinum-based chemotherapy and immune checkpoint blockade) by the U.S. Food and Drug Administration in December 2019, followed by its approval by the European Medicines Agency in 2022. Interestingly, *TP53* mutations are mutually exclusive with *FGFR3* mutations in conventional bladder or upper tract UC [22,23,24]. However, p53 and FGFR3 protein expression, which could be a more clinically applicable diagnostic tool, has not been systematically examined. Furthermore, no studies have investigated whether p53 and FGFR3 expression is associated with oncologic outcomes in patients with mUC receiving EV. We therefore immunohistochemically assessed the relative expression of p53 and FGFR3 in surgical specimens from those with mUC and correlated their status with response to EV therapy.

## 2. Results

### 2.1. Patient Characteristics

We immunohistochemically evaluated 28 UC specimens for the expression of p53, FGFR3, and nectin-4. The baseline characteristics of the 28 patients with mUC who received EV, including 18 (64.3%) with pure conventional UC, are summarized in Table 1. The median age was 74 years and most were males (89.3%). As for the primary tumor site, 13 (46.4%) and 15 (53.6%) were in the bladder and upper tract, respectively. Histologically, 10 (35.7%) patients showed conventional UC along with its variants, including squamous differentiation (n = 6; 21.4%), glandular differentiation (n = 1; 3.6%), trophoblastic differentiation (n = 1; 3.6%), sarcomatoid differentiation (n = 1; 3.6%), or small cell neuroendocrine carcinoma (n = 1; 3.6%). Six (21.4%) patients had an Eastern Cooperative Oncology Group performance status [25] of ≥2, and 11 (39.3%) had Bellmunt risk factors [26] of ≥2. The number of systemic therapy lines administered before EV therapy was two in 22 (78.6%) patients and three or more in 6 (21.4%) patients. Prior immunotherapy included avelumab (n = 11; 39.3%) and pembrolizumab (n = 17; 60.7%).

### 2.2. p53/FGFR3/Nectin-4 Immunohistochemistry (IHC) and Response to EV Treatment

**Figure 1:**
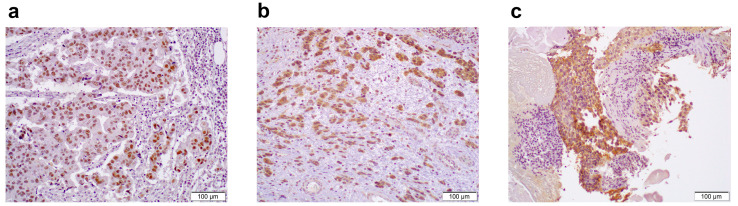
Immunohistochemistry of p53, FGFR3, and nectin-4 in high-grade urothelial carcinoma. (**a**) A case of pure conventional urothelial carcinoma, where a *TP53* missense mutation and wild-type *FGFR3* were harbored and complete response to enfortumab vedotin was achieved, showing diffuse nuclear p53 expression. (**b**) A case of pure conventional urothelial carcinoma, where an *FGFR3* gain-of-function mutation and a wild-type *TP53* were harbored and where disease progression was seen after enfortumab vedotin therapy, showing strong cytoplasmic FGFR3 expression. (**c**) A case of pure conventional urothelial carcinoma, where partial response to enfortumab vedotin was seen, showing strong membranous nectin-4 expression. Original magnification: 200×.

We defined nuclear p53 +1 (positive in <50% of tumor cells) as a wild-type pattern, while 0 or +2 (positive in ≥50% tumor cells) as an abnormal pattern, as described in the Materials and Methods section. An abnormal p53 staining pattern was detected in 17 (60.7%) of all cases, and 11 (61.1%) of pure UC cases, including 1 (5.6%) with null staining (Figure 1a). High FGFR3 expression was seen in 5 (17.9%) of all cases and 4 (22.2%) of the pure UC cases (Figure 1b). When considering the expression status of both p53 and FGFR3, the results are summarized in Table 2. A total of 14 (50.0%) of all cases and 9 (50.0%) of the pure UC cases showed abnormal p53/low FGFR3, whereas 2 (7.1%) of all cases and 2 (11.1%) of the pure UC cases showed wild-type p53/high FGFR3. For the combination of p53 and FGFR3, the wild-type p53 and low FGFR3 (double-negative)/abnormal p53 and high FGFR3 (double-positive) groups included 9/3 (32.1%/10.7%) of all cases and 5/2 (27.8%/11.1%) of the pure UC cases, respectively. The median H-scores of nectin-4 were 60 in all patients and 70 in the pure UC patients (Figure 1c). The expression of nectin-4 (high vs. low) was not significantly associated with that of p53 (*p* = 0.638; in all cases, *p* = 1.000; in the pure UC cases). By contrast, the expression of nectin-4 showed significant association with that of FGFR3 (*p* = 0.031; in all cases, *p* = 0.049; in the pure UC cases).

Genomic data were available for 7 (25.0%) patients. These included 3 (of 4 with abnormal p53 expression) harboring a *TP53* missense mutation and 3 (of 3 with 1+ p53) having wild-type *TP53* (n = 2) and a splice variant (unknown impact on p53 expression; n = 1). Similarly, 1 patient with high FGFR3 expression harbored an *FGFR3* gain-of-function mutation, and the other 6 showing low FGFR3 had wild-type *FGFR3*.

The best overall response to EV in all cases (Table 3) and pure UC cases (Table 4) categorized by the status of p53 and/or FGFR3 immunostaining are shown. In all cases, objective response rates (ORR) (*p* = 0.038) and disease control rates (DCR) (*p* = 0.033) were significantly higher in patients with abnormal p53 than in those with wild-type p53. Of note, three patients with abnormal p53 expression achieved complete response (CR). FGFR3 IHC showed no statically significant difference in response to EV treatment in all cases, while the pure UC patients with low FGFR3 showed better ORR than those with high FGFR3. When combining p53 and FGFR3, abnormal p53/low FGFR3 (vs. wild-type p53/high FGFR3) was associated with significantly better response in both all cases (*p* = 0.002) and the pure UC cases (*p* = 0.023) categories. Additionally, in the entire cohort, patients with abnormal p53/low FGFR3 (vs. wild-type p53/high FGFR3) had better DCR (*p* = 0.013). Moreover, both of the two pure UC patients with wild-type-p53/high FGFR3 showed progressive disease (PD) after EV treatment. Nectin-4 expression dichotomized at the median score showed no statically significant difference in response to EV treatment (*p* = 0.085 in the entire cohort; *p* = 0.159 in the pure UC cases).

### 2.3. p53/FGFR3/Nectin-4 IHC and Response to EV Treatment in UC with Histological Variants

In six patients with squamous differentiation, five (83.3%) and one (16.7%) demonstrated abnormal p53/low FGFR3 and abnormal p53/high FGFR3 expression, respectively. High nectin-4 expression was seen in three (50%) patients with squamous differentiation. The patients with glandular differentiation, trophoblastic differentiation, sarcomatoid differentiation, or small cell neuroendocrine carcinoma showed wild-type p53/low FGFR3 and low nectin-4 expression. The objective responses to EV treatment in patients with histological variants was as follows: partial response (PR) in six of six cases with squamous differentiation; stable disease (SD) in a case with glandular differentiation; PD in a case with sarcomatoid differentiation; and SD in a case with small cell neuroendocrine carcinoma.

### 2.4. Relation between p53/FGFR3/Nectin-4 and Survival

**Figure 2:**
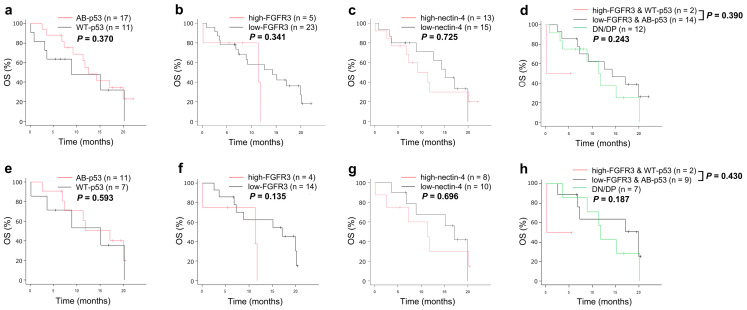
Kaplan–Meier curves for OS in patients with UC (**a**–**d**) or pure UC (**e**–**h**), according to the expression status of p53 and/or FGFR3, or nectin-4. Abbreviations: OS, overall survival; AB, abnormal; WT, wild-type; DN/DP, double-negative/double-positive.

The median overall survival (OS) was 12.6 months (95% confidence interval = 7.3–20.0). The relationship between IHC data and actual patient survival was then assessed. A Kaplan-Meier analysis for OS revealed no significant differences according to the status of p53, FGFR3, nectin-4, or a combination of p53 and FGFR3 in both the entire cohort and the pure UC group (Figure 2).

## 3. Discussion

We have provided data for the first time on the relationship between p53/FGFR3 immunostaining status and response to EV treatment in patients with mUC. In this immunohistochemical study, the expression status of p53 and FGFR3 was associated with response to EV treatment. Remarkably, the combination of p53 and FGFR3 expression reflected response in both all cases and pure UC cases.

The *TP53* gene encodes for the p53 protein, a tumor suppressor that plays a crucial role in the development and progression of UC. *TP53* is one of the most commonly altered genes in UC and is associated with poor oncologic outcomes [14]. Based on TCGA exome data, *TP53* is the most frequently mutated gene for the Ba/Sq subtype (61%, *p*-adjusted = 0.002) [27], which is the most frequent molecular class and is associated with poor survival [19]. Abnormal p53 expression, defined as 0% or ≥50% nuclear staining, was detected in 59% of high-grade UCs treated uniformly with cystectomy [28], and was associated with poor recurrence-free survival [28]. In addition, p53 deficiency or inactivation is a known mechanism of drug resistance in human malignancies [29]. Nevertheless, to the best of our knowledge, no studies have indicated the implications of p53 protein status on the efficacy of EV treatment. However, p53 is known to play a crucial role in DNA repair [30]. Moreover, it has been documented that monomethyl auristatin E, a composition of EV, disrupts microtubule formation, which results in cell-cycle arrest and apoptosis [4]. These findings might partially explain the mechanisms behind why an abnormal p53 staining was associated with better response to EV treatment. A next-generation sequencing study showed *TP53* or *MDM2* somatic alterations as potential biomarkers of response to EV in patients with advanced UC [31]. In the present study, UC patients with abnormal p53 expression had significantly a better response rate to EV. Nonetheless, p53 abnormality is known to be a negative prognostic biomarker, and our results showed no significant difference in OS between patients with abnormal p53 and wild-type p53 expression. Abnormal p53 IHC expression reflects mutations in not only *TP53* but also in *MDM2*, because *MDM2* alterations lead to a reduction or loss of function of p53 [31]. Again, this is the first study demonstrating the association between p53 immunohistochemical expression and outcomes in patients with mUC receiving EV. These findings, if validated, may importantly facilitate patient selection for EV treatment.

FGFR3 is encoded by the *FGFR3* oncogenic gene, whose genomic alterations are potent oncogenic drivers in UC [19] and represent predictive biomarkers of response to FGFR inhibitors, such as erdafitinib [22]. *FGFR3* alterations occur in 12% of muscle-invasive bladder cancers [14], 22% of advanced UC or mUC cases [20], and 36% of high-grade upper tract UC cases [20]. Notably, *FGFR3* mutations promote resistance to platinum-based chemotherapy and immunotherapy. The luminal-papillary (TCGA group) subtype characterized by *FGFR3* mutations showed a low likelihood of response to platinum-based neoadjuvant chemotherapy [15]. The luminal I subtype, which is associated with a poor response to immune checkpoint blockade [17,18], shows a relatively lower immune signature and lower expression of programmed death ligand 1 in tumors and associated immune cells [32] than other subtypes, as well as a higher percentage of *FGFR* mutations [32]. In the present study, pure UC patients with high FGFR3 revealed significantly lower ORR to EV treatment than those with low FGFR3. Interestingly, *FGFR3* and *TP53* mutations are mutually exclusive in high-grade UCs, especially in non-invasive tumors [33], whereas *TP53*-mutated muscle-invasive bladder cancers may possibly harbor *FGFR3* mutations [14]. In our data, three (10.7%) of all cases and two (11.1%) of the pure UC cases showed abnormal p53 and high FGFR3 expression. Remarkably, the combination of p53 and FGFR3 expression status, as a useful biomarker, predicted response to EV treatment in patients with mUC.

In daily clinical practice, conducting a whole genome profiling test on every patient might be too expensive and time-consuming. The subtyping of UC using a small number of immunohistochemical markers, including p53 for basal tumors and FGFR3 for luminal tumors, is regarded as an alternative since IHC is a relatively inexpensive, readily available, and reliable method.

There are several limitations to this study. First, the study was retrospective, with a relatively small sample size in our cohort. Accordingly, our results might be confounded by unobserved factors and might also need to be interpreted with caution. Second, the follow-up duration of the cohort was relatively short. Third, our cohort included both bladder cancer (n = 13) and upper urinary tract cancer (n = 15) cases. Although these cancers arise from the urothelium and are histologically similar, they may now be considered distinct entities with different pathways involving their carcinogenesis [34]. Finally, the optimal scoring and cut-off values for p53 and FGFR3 IHC need to be investigated in a validation cohort. Large-scale, prospective studies are warranted to validate our results.

## 4. Materials and Methods

### 4.1. Patient Population

This study retrospectively evaluated 45 consecutive patients with mUC who had received EV after the failure of platinum-based chemotherapy and immunotherapy (avelumab or pembrolizumab) at the University of Occupational and Environmental Health (UOEH) between December 2021 and March 2024. All patients were histologically diagnosed with UC of the bladder or upper tract, with or without determining histological variants, and showed radiologically confirmed disease progression as metastatic disease. The exclusion criteria were treatment with neoadjuvant chemotherapy or radiation therapy prior to tissue collection (n = 2) and unavailable tissues for IHC (n = 15).

### 4.2. Patient Management

EV was administered as an intravenous infusion at a dose of 1.25 mg/kg on days 1, 8, and 15 of a 28-day cycle. EV treatment continued until disease progression, unacceptable adverse events, or consent withdrawal. Routine follow-up consisted of physical examinations, laboratory tests, and chest–abdominal–pelvic computed tomography. Computed tomography was conducted at baseline and after every 1–3 cycles of EV. In some of the patients, tumor genomic profiling, including *TP53* and *FGFR3* alterations, was performed using validated, hybrid-capture-based next-generation sequencing assays (FoundationOne^®^ CDx test).

The objective response to EV treatment was assessed according to the Response Evaluation of Criteria in Solid Tumours, version 1.1 [35]. ORR was defined as the proportion of patients with CR and PR, but not SD and PD. DCR comprised ORR and SD.

### 4.3. Immunohistochemical Staining

IHC was carried out as we described previously [36]. Briefly, staining was performed on the sections (4 µm thick) using a primary antibody targeting p53 (clone B-P3, dilution 1:50, Santa Cruz Biotechnology, Dallas, TX, USA), FGFR3 (clone B-9, dilution 1:50, Santa Cruz Biotechnology), or nectin-4 (clone EPR15613-68, dilution 1:100, Abcam, Cambridge, UK), followed by the secondary antibody-peroxidase-linked polymers. The p53 antibody could react with an epitope between residues 16 and 25 and detect both wild-type and mutant p53 proteins. Appropriate positive controls for the stains (e.g., skin, uterine serous carcinoma) and negative tissue elements were evaluated and were adequate for the interpretation in UC. All stains were manually quantified by an experienced and board-certified pathologist (H.M.) who was blinded to sample identification.

### 4.4. Scoring of IHC

Nuclear p53 expression was scored as 0 (negative), +1 (positive in <50% of tumor cells), and +2 (positive in ≥50% tumor cells) [24,28,37]. p53 +1 was categorized as a wild-type pattern, while 0 or 2+ was categorized as an abnormal pattern because a strong and diffuse pattern and a complete absence are commonly associated with *TP53* missense and nonsense mutations, respectively [24,37,38]. For FGFR3, the German immunoreactive scores (range: 0–12) calculated by multiplying the percentage of immunoreactive cells (0% = 0; 1–10% = 1; 11–50% = 2; 51–80% = 3; 81–100% = 4) by staining intensity (negative = 0; weak = 1; moderate = 2; strong = 3) were considered negative (0; 0–1), weakly positive (1+; 2–4), moderately positive (2+; 6–8), and strongly positive (3+; 9–12). FGFR3 expression was then dichotomized into low (0/+1) and high (2+/3+). For nectin-4 IHC, cytoplasmic and membranous staining in tumor cells was scored using the H-score system, which is the product of intensity (score, 0–3) and percentage of stained cells (0–100), as used in the initial study of nectin-4 [39].

### 4.5. Statistical Analysis

All statistical analyses were carried out using EZR version 1.65 (Easy R, Vienna, Austria) [40], a graphical user interface for R (The R Foundation for Statistical Computing). The Chi-square test was used to evaluate the associations between categorized variables in two groups. One-way ANOVA with Holm’s post hoc test was used for comparison of three groups. OS was defined as the interval from EV initiation to all-cause death or last follow-up in patients without death. The Kaplan-Meier method was conducted to calculate survival rates, and the log-rank test was used for comparisons. *p*-values of less than 0.05 were considered statistically significant.

## 5. Conclusions

The present results indicate that the immunohistochemical expression of p53 and FGFR3 may precisely predict response to EV therapy in patients with UC, which may further provide biomarker-driven, personalized therapeutic approaches for UC. External and prospective validation of these findings is needed in larger cohorts.

## Figures and Tables

**Table 1:** Patient characteristics.

Characteristics	All Patients (n = 28)	Patients with PUC (n = 18)
Age (years), median (IQR)	74 (66–79)	73 (64–75)
Sex, n (%)		
Male	25 (89.3)	16 (88.9)
Female	3 (10.7)	2 (11.1)
ECOG-PS score, n (%)		
0	13 (46.4)	9 (50.0)
1	9 (32.1)	4 (22.2)
2	5 (17.9)	4 (22.2)
3	1 (3.6)	1 (5.6)
Primary tumor site, n (%)		
Bladder	13 (46.4)	8 (44.4)
Upper urinary tract	15 (53.6)	10 (55.6)
Metastatic lesion, n (%)		
Lymph node	22 (78.6)	15 (83.3)
Lung	11 (39.3)	7 (38.9)
Liver	4 (14.3)	2 (11.1)
Bone	5 (17.9)	3 (16.7)
Bellmunt risk score, n (%)		
0, 1	17 (60.7)	11 (61.1)
≥2	11 (39.3)	7 (38.9)
Number of prior lines of systemic therapy, n (%)		
2	22 (78.6)	13 (72.2)
≥3	6 (21.4)	5 (27.8)
Prior systemic chemotherapy, n (%)		
Gemcitabine + cisplatin	13 (46.4)	9 (50.0)
Gemcitabine + carboplatin	12 (42.9)	7 (38.9)
Paclitaxel + gemcitabine	8 (28.6)	7 (38.9)
Dose-dense MVAC	1 (3.6)	1 (5.6)
Cisplatin + etoposide	1 (3.6)	0 (0.0)
Prior immune checkpoint blockade, n (%)		
Avelumab	11 (39.3)	8 (44.4)
Pembrolizumab	17 (60.7)	10 (55.6)
EV cycles, median (IQR)	5 (4–8)	6 (4–10)
Follow-up duration (months), median (IQR)	9 (6–17)	12 (7–18)

IQR, interquartile range; ECOG-PS, Eastern Cooperative Oncology Group performance status; MVAC, methotrexate, vinblastine, doxorubicin, and cisplatin; EV, enfortumab vedotin; PUC, pure urothelial carcinoma.

**Table 2:** p53/FGFR3 expression patterns in patients with UC or PUC.

In All (n = 28)			In PUC (n = 18)		
No. %	WT-p53	AB-p53	No. %	WT-p53	AB-p53
**low FGFR3**	14 (50.0)	9 (32.1)	**low FGFR3**	9 (50.0)	5 (27.8)
**high FGFR3**	3 (10.7)	2 (7.1)	**high FGFR3**	2 (11.1)	2 (11.1)

PUC, pure urothelial carcinoma; WT, wild-type; AB, abnormal.

**Table 3:** Observed efficacy of enfortumab vedotin in all patients stratified by p53 and FGFR3 expression.

Response in All (n = 28)	WT p53	AB p53	*p* Value	Low FGFR3	High FGFR3	*p* Value	Response in All (n = 28)	AB p53 & Low FGFR3	WT p53 & High FGFR3	DN/DP	*p* Value
	No. %	No. %		No. %	No. %			No. %	No. %	No. %	
Best response			**0.024**			0.126	Best response				**0.001**
CR	0 (0.0)	3 (10.7)		3 (10.7)	0 (0.0)		CR	3 (10.7)	0 (0.0)	0 (0.0)	
PR	4 (14.3)	11 (39.3)		14 (50.0)	1 (3.6)		PR	10 (35.7)	0 (0.0)	5 (17.9)	
SD	3 (10.7)	3 (10.7)		4 (14.3)	2 (7.1)		SD	1 (3.6)	0 (0.0)	5 (17.9)	
PD	4 (14.3)	0 (0.0)		2 (7.1)	2 (7.1)		PD	0 (0.0)	2 (7.1)	2 (7.1)	
ORR	4 (14.3)	14 (50.0)	**0.038**	17 (60.7)	1 (3.6)	0.078	ORR	13 (46.4)	0 (0.0)	5 (17.9)	**0.004**
DCR	7 (25.0)	17 (60.7)	**0.033**	21 (75.0)	3 (10.7)	0.268	DCR	14 (50.0)	0 (0.0)	10 (35.7)	**<0.001**

CR, complete response; PR, partial response; SD, stable disease; PD, progressive disease; ORR, objective response rate; DCR, disease control rate; WT, wild-type; AB, abnormal; DN/DP, double-negative or double-positive.

**Table 4:** Observed efficacy of enfortumab vedotin in patients with pure UC stratified by p53 and FGFR3 expression.

Responsein PUC (n = 18)	WT p53	AB p53	*p* Value	Low FGFR3	High FGFR3	*p* Value	Responsein PUC (n = 18)	AB p53 & Low FGFR3	WT p53 & High FGFR3	DN/DP	*p* Value
	No. %	No. %		No. %	No. %			No. %	No. %	No. %	
Best response			0.073			**0.039**	Best response				**0.012**
CR	0 (0.0)	3 (16.7)		3 (16.7)	0 (0.0)		CR	3 (16.7)	0 (0.0)	0 (0.0)	
PR	3 (16.7)	5 (27.8)		8 (44.4)	0 (0.0)		PR	5 (27.8)	0 (0.0)	3 (16.7)	
SD	1 (5.6)	3 (16.7)		2 (11.1)	2 (11.1)		SD	1 (5.6)	0 (0.0)	3 (16.7)	
PD	3 (16.7)	0 (0.0)		1 (5.6)	2 (11.1)		PD	0 (0.0)	2 (11.1)	1 (5.6)	
ORR	3 (16.7)	8 (44.4)	0.441	11 (61.1)	0 (0.0)	**0.024**	ORR	8 (44.4)	0 (0.0)	3 (16.7)	**0.030**
DCR	4 (22.2)	11 (61.1)	0.084	13 (72.2)	2 (11.1)	0.205	DCR	9 (50.0)	0 (0.0)	6 (33.3)	**0.003**

PUC, pure urothelial carcinoma; CR, complete response; PR, partial response; SD, stable disease; PD, progressive disease; ORR, objective response rate; DCR, disease control rate; WT, wild-type; AB, abnormal; DN/DP, double-negative or double-positive.

## Data Availability

The data presented in this study are available on request from the corresponding authors but are not publicly available due to privacy and/or ethical restrictions.
